# Malaria: Factors affecting disease severity, immune evasion mechanisms, and reversal of immune inhibition to enhance vaccine efficacy

**DOI:** 10.1371/journal.ppat.1012853

**Published:** 2025-01-23

**Authors:** Xin-zhuan Su, Fangzheng Xu, Rachel V. Stadler, Awet Alem Teklemichael, Jian Wu

**Affiliations:** Malaria Functional Genomics Section, Laboratory of Malaria and Vector Research, National Institute of Allergy and Infectious Disease, National Institutes of Health, Rockville, Maryland, United States of America; University of Utah, UNITED STATES OF AMERICA

## Abstract

Malaria is a complex parasitic disease caused by species of *Plasmodium* parasites. Infection with the parasites can lead to a spectrum of symptoms and disease severity, influenced by various parasite, host, and environmental factors. There have been some successes in developing vaccines against the disease recently, but the vaccine efficacies require improvement. Some issues associated with the difficulties in developing a sterile vaccine include high antigenic diversity, switching expression of the immune targets, and inhibition of immune pathways. Current vaccine research focuses on identifying conserved and protective epitopes, developing multivalent vaccines (including the whole parasite), and using more powerful adjuvants. However, overcoming the systematic immune inhibition and immune cell dysfunction/exhaustion may be required before high titers of protective antibodies can be achieved. Increased expression of surface molecules such as CD86 and MHC II on antigen-presenting cells and blocking immune checkpoint pathways (interactions of PD-1 and PD-L1; CTLA-4 and CD80) using small molecules could be a promising approach for enhancing vaccine efficacy. This assay reviews the factors affecting the disease severity, the genetics of host–parasite interaction, immune evasion mechanisms, and approaches potentially to improve host immune response for vaccine development.

## 1. Malaria and parasite life cycle

**Fig 1:**
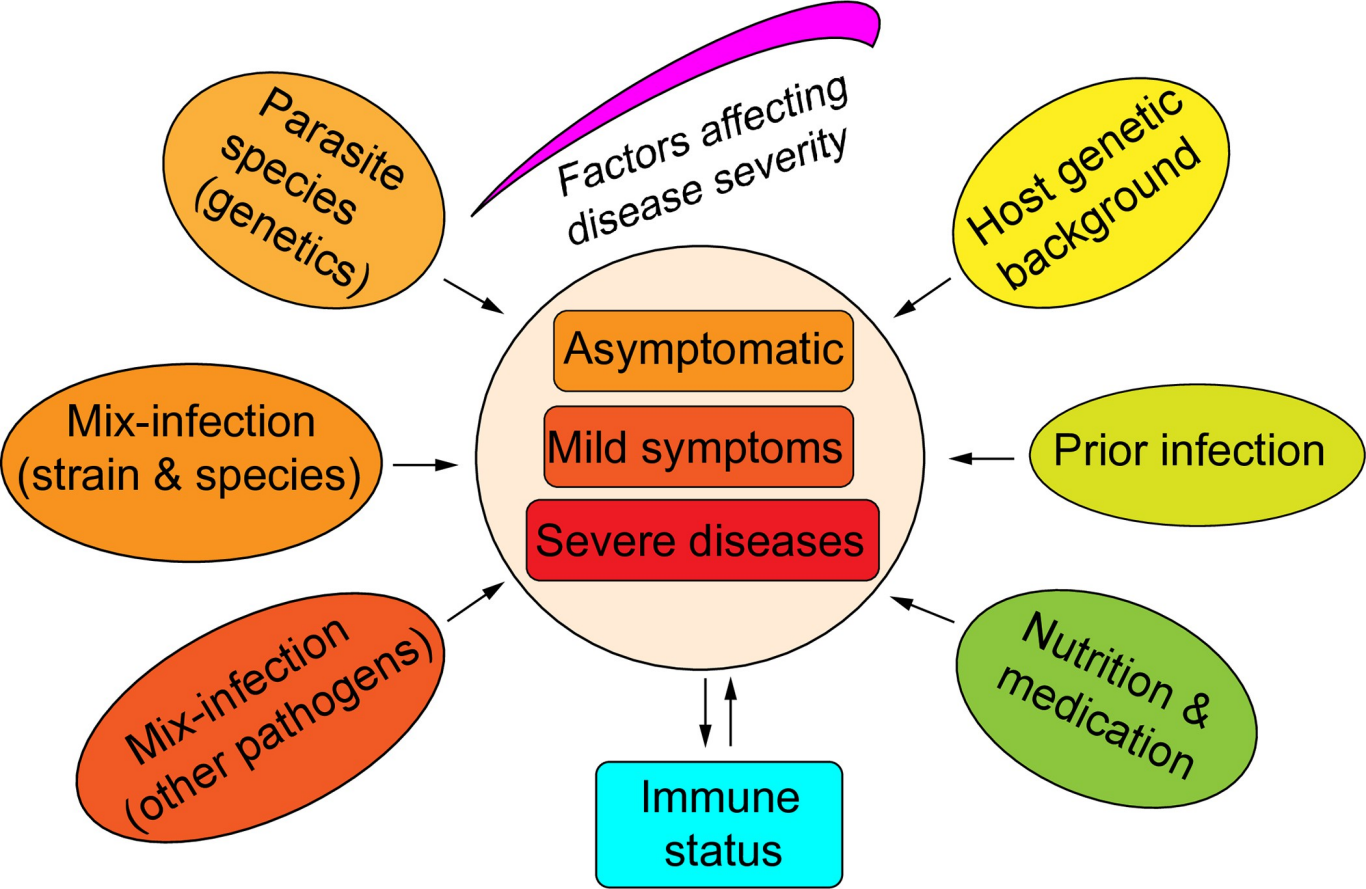
Factors that can influence malaria disease severity. Parasite factors include molecules mediating the invasion of RBCs and modulating host immune responses. Host genetic background can influence parasite invasion of RBCs and host immunity, too. Prior infections, nutritional status, and anti-malaria treatment also contribute to disease severity.

**Fig 2:**
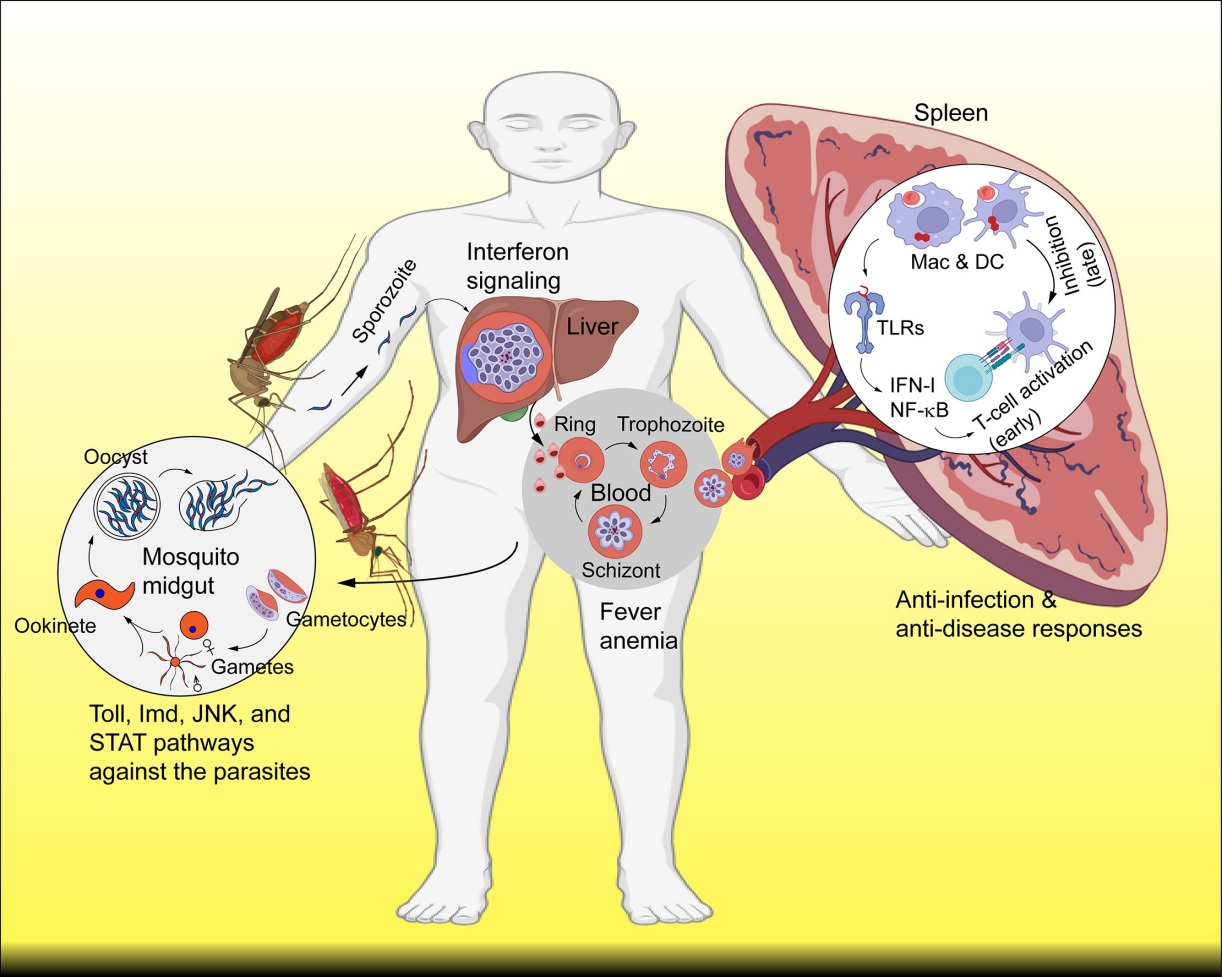
Many parasite life cycle stages can trigger host immune responses. Sporozoites are injected into the human skin when an infected mosquito bites a human host. The parasites migrate to the liver, developing into schizonts containing thousands of merozoites. Merozoites are released into the blood and invade RBCs, within which they develop from rings to trophozoites to schizonts. Mature schizonts again release merozoites to infect more RBCs. Some of the merozoites differentiate into male and female gametocytes. When another mosquito takes blood, the male and female gametocytes fertilize to produce zygotes that differentiate into ookinetes and oocysts. Sporozoites within oocysts migrate to the salivary glands of the mosquito. When the mosquito bites another human host, the sporozoites will start a new cycle. iRBCs, parasite nucleic acids, and metabolites can be picked up by DCs and macrophages (Mac) to trigger immune responses through activation of Toll-like (TLR) and other receptors in immune organs such as the spleen or lymph nodes in early infection. Activation of DCs and other antigen-presenting cells will activate T and B cells, leading to antibody production later. Parasite materials such as hemozoin could also induce immune cell exhaustion and immune inhibition later in the infection. Other immune mechanisms are also activated, such as interferon signaling against liver stages and the Toll, Imd, JNK, and STAT pathways to kill mosquito stages. Some images of cells and receptors were adopted from BioRender.

We typically describe malaria as a life-threatening disease caused by *Plasmodium* parasites through the bites of infected female *Anopheles* mosquitoes. However, malaria is not a single disease in many ways, and many factors can influence disease outcomes (**Fig 1**). First, humans can be infected by 6 *Plasmodium* species, including *Plasmodium falciparum*, *Plasmodium vivax*, *Plasmodium malariae*, *Plasmodium knowlesi*, *Plasmodium cynomolgi*, and *Plasmodium ovale*, which has 2 subspecies, *P*. *o*. *cutisi* and *P*. *o*. *wallikeri*. Although infections with malaria parasites result in some common symptoms (fever and anemia), each parasite species or strain can cause specific disease phenotypes. Second, mix-infections of more than one malaria parasite species or strains and co-infections with other pathogens can alter disease progression and severity. Third, the host’s genetic background plays a significant role in the outcome of an infection. Fourth, the strain, timing, and number of prior exposures (immune status) may determine the level and direction of the host immune response. Fifth, host nutritional status and administration of anti-malarial drugs can also influence disease outcomes. Indeed, individuals infected with malaria parasites may be asymptomatic, can be mild with fever, or develop into a disease with severe anemia, respiratory distress, metabolic acidosis, cerebral malaria, and multi-organ failure leading to death. All malaria parasite species have similar life cycles and developmental stages, although the time to complete a life cycle is species specific (**Fig 2**).

## 2. Parasite genetic factors affecting disease outcomes

Both host and parasite populations are genetically complex, and the interaction of the complex genomes can influence disease outcomes. Dissecting the molecular mechanism of host–parasite interaction and a better understanding of the roles of host and parasite genetic factors during an infection will provide critical information for disease management and control.

### Malaria parasite species and disease severity

Different parasite species inherently cause diseases with various symptoms or disease severity. For instance, most malaria-related deaths are caused by *P*. *falciparum*, while *P*. *vivax* infection is generally nonlethal, underscoring the crucial role of parasite genetics and biology in disease manifestation. *P*. *vivax* only invades reticulocytes and produces hypnozoites, which can cause relapses in the liver, whereas *P*. *falciparum* can infect all red blood cell (RBC) types and does not cause relapses. The selective invasion of reticulocytes by *P*. *vivax* could contribute to the relatively “mild” disease because the parasites will grow slowly in searching for a host cell suitable for invasion, which may give the host more time to produce antibodies to control the infection.

### Genetic differences within a species

Infection with different *P*. *falciparum* strains can also contribute to the variation in disease manifestation. Infections of different *P*. *falciparum* strains or parasites carrying specific antigenic alleles were associated with parasite density and disease severity. For example, the merozoite surface protein 1 (MSP-1) allelic family MSP1-MAD20 was associated with high parasitemia [1]. Infections with parasites carrying MSP-1 RO33/K1 alleles were associated with fever, independent of age and parasite density [2].

### Mixed species/strain infections

In malaria-endemic regions, patients have a high frequency of multi-strain infection (30% to 60% of the patients) [3]. Patients with mixed species infection had a higher proportion of severe anemia, pulmonary complications, and multiple organ failure than those with *P*. *falciparum* mono-infection [4]. The complexity of the FC27 genotypes was significantly higher in 405 children with severe malaria in Boulgou, Burkina Faso [5]. Therefore, the number of strains in a *P*. *falciparum* infection correlates with increased severity.

### Co-infection with other pathogens

A malaria patient may be co-infected with other disease agents, including other parasite species, fungi, bacteria, and viruses. These pathogens can interact with each other and the host immune system to influence disease outcomes. Co-infection of mice with *Trypanosoma brucei* and *Plasmodium berghei* increased the number of both parasites, leading to more severe anemia, hypoglycemia, and lower survival [6]. Helminth infections have been reported to increase or decrease susceptibility against *Plasmodium* infections in animal models and human populations [7]. Patients with malaria appeared at greater risk of bacteremia and death, although the prevalence of co-infection was low [8].

### Diverse disease pathologies and virulence from rodent parasite species and strains

Parasite genetics affecting malaria disease phenotypes have clearly been demonstrated using the rodent malaria parasite *Plasmodium yoelii*. Inbred mice can be infected with parasite strains having diverse or similar genetic backgrounds, and the disease symptoms or severity can be compared, providing direct evidence of the effect of parasite genetic variation on disease phenotypes. *P*. *yoelii* strains 17XNL, 17XL, and YM were derived from 17X during propagation in different laboratories. However, 17XL and YM are lethal, killing C57BL/6j mice in approximately 7 days after injections of 1 × 10^6^ infected RBCs, whereas 17X and 17XNL are nonlethal and are cleared by the host day 20 postinfection (pi) [9]. The YM (17XL) parasites grow fast and likely kill their hosts through severe anemia. An amino acid (C713R) substitution in domain 6 of the *P*. *yoelii* erythrocyte-binding-like (PyEBL) altered parasite growth rate and disease phenotype [10]. Similarly, subspecies *Plasmodium y*. *nigeriensis* N67 and N67C are isogenic strains. N67C kills the host in 7 days due to a high level of inflammation, but mice infected with N67 can survive for 20 days [9]. Again, another amino acid substitution in the domain 6 (C741Y) of the PyEBL changed the parasite growth, host immune responses, and disease phenotypes [11]. A single amino acid change (S to F) in the *Plasmodium berghei* (Pb) NK65 ApiAP2 gene resulted in early IFN-γ responses and high levels of IgG2b and IgG2c antibodies [12].

Co-infections of mice with *P*. *yoelii* and *Plasmodium vinckei* or *Plasmodium chabaudi* increased virulence, leading to 100% mortality, compared to no mortality for single infections of *P*. *yoelii* CU strain or *P*. *vinckei* DS strain and 40% mortality for *P*. *chabaudi* AJ single infections [13]. Virulent *P*. *chabaudi* clones had a competitive advantage over nonvirulent clones in the acute phase of mixed infections [14]. Indeed, evolution modeling of rodent and human malaria infections showed strong positive correlations between asexual multiplication, transmission rate, infection length, morbidity, and mortality and predicted parasite populations evolving to new levels of virulence in response to vaccines and drugs [15].

## 3. Host factors affecting disease progression and severity

Many host factors can affect the efficiencies of parasite invasion of RBCs and host immunity. Understanding the effects of these factors on disease outcomes will help us design better laboratory experiments and clinical studies.

### Host genetic variants affecting parasite invasion and growth

Host genetic variants can confer resistance to malaria infection. The Duffy Antigen Receptor for Chemokines (DARC) is expressed on the surface of RBCs, and *P*. *vivax* uses DARC to invade RBCs [16]. DARC-negative human erythrocytes are resistant to invasion by *P*. *vivax*, although complement receptor 1 (CR1) was recently shown to be another receptor for *P*. *vivax* invasion [17]. Similarly, *P*. *falciparum* erythrocyte binding antigen 175 (PfEBA-175) and *P*. *falciparum* MSP-1 can bind glycophorin A (GYPA) for invasion [18]. For additional host molecules that can impact parasite growth, invasion of RBCs, and host–parasite interaction, readers can consult the reviews published previously [19,20].

### Host immune molecules affecting parasite growth and disease severity

The host’s genetic background can affect the level and direction of immune response to malaria parasite infections. For example, a Toll-like receptor 2 (TLR2) Δ22 polymorphism was associated with protection from cerebral malaria in a case-control study [21]. Proinflammatory cytokines such as IL-1, IL-6, IL-8, IL-12, IFN-γ, and TNF-α are critical for controlling *Plasmodium* infection, and the plasma IL-10:TNF-α ratio was associated with TNF promoter variants and could predict malarial complications [22]. Genetic variations in the IFN-γ gene were also associated with cerebral malaria (CM), suggesting that IFN-γ protects against CM through anti-parasite activity [23].

### The timing of infection and cytokine dynamics

When parasites are injected, the host mounts an innate response with proinflammatory cytokine production and immune cell activation to control pathogen replication. As the infection progresses, the host responses turn into anti-inflammatory or anti-disease responses by producing IL-10 and induction of regulatory subsets of innate immune cells (immunoregulation) [24]. Levels of cytokines are dynamic, typically with an increase in early infection and decrease gradually as the infection progresses. For example, most cytokines and chemokines peaked days 4 to 7 postinjection of *P*. *yoelii* 17XNL infected red blood cells (iRBCs) and declined to baseline levels on day 10 postinjection [25], suggesting a state of immune suppression. In clinical studies, blood samples were generally collected from patients without knowing the time of infection or disease progression status. The readouts between individual clinical samples may not be comparable due to the timing of infection and the dynamic responses to malaria parasite infections and could present problems for comparative or association studies.

### Prior exposure and persistent parasitemia on host immune capability

Previous exposure history can be a significant factor in determining disease outcomes and vaccination efficacy. Multiple episodes of infection or persistent blood-stage parasitemia will induce anti-disease immunity that can inhibit anti-infection immune response and negatively affect vaccine-induced protection [24,26]. Repeated *Plasmodium* infections will suppress protective inflammatory responses, which may also interfere with the ability to generate suitable protective immunity, including producing high titers of antibodies [27].

## 4. Mechanisms of immune inhibition and evasion

**Fig 3:**
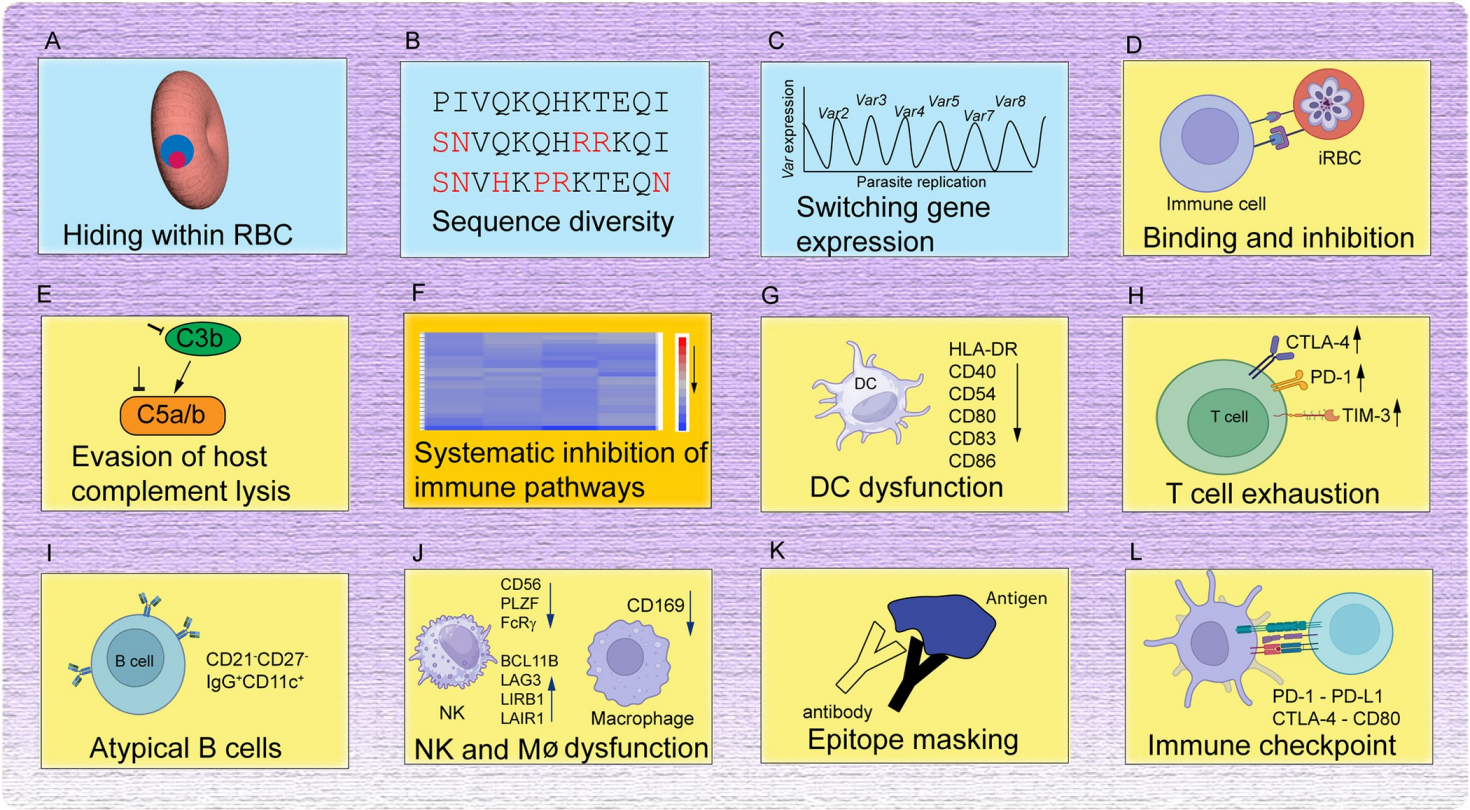
Major mechanisms of immune evasion in malaria. (**A**) Parasite lives within RBCs, avoiding various intracellular killing mechanisms, although parasite proteins expressed on the surface of RBCs can be recognized by the host immune system. (**B**) Parasite molecules exposed to host immunity are highly polymorphic, allowing survival under strain-specific immunity. (**C**) Expression of a different copy of a variant antigen gene (*var*) can also evade immunity against a previously expressed variant. (**D**) Binding of parasite proteins such as PfEMP1 and PfRIFIN to various host cells can induce immune inhibition and avoid clearance by the spleen. (**E**) Binding of parasite proteins to molecules in the host complement system blocks complement-mediated lysis and killing of the parasites. (**F**) Genome-wide transcriptional analyses show down-regulation of host genes in many immune pathways, including B and T cell activations. (**G**) DC dysfunction with reduced expression of many surface proteins required for T cell activation. (**H**) Malaria parasite infection also leads to T cell dysfunction and exhaustion with increased expression of T cell exhaustion and senescence markers. (**I**) Expansion of a group of B cells with reduced B cell receptor signaling was recognized in malaria. The status and functions of the atypical B cell required further investigation. (**J**) Dysfunction of macrophages and NK cells was also observed in malaria. Dysfunction of erythropoietic island macrophage can also cause anemia. (**K**) Antibodies from prior exposure or immunization may block the binding of new antibodies generated. (**L**) Many immune checkpoint pathways are activated in malaria, and blockade of the pathways can reverse immune inhibition, inhibit parasite growth, and enhance host survival in rodent malaria models. Note that many of these elements are overlapping and can be integrated into a broad systematic immune inhibition. Some images of cells and receptors were adopted from BioRender.

Malaria parasites developed various immune escape strategies to survive within their hosts [28,29]. One advantage of the blood stages of malaria parasites is that RBCs do not have a nucleus and lack the expression of immune genes. Thus, the parasites can avoid a direct attack from intracellular defense mechanisms such as autophagy or recognition of pathogen recognition receptors within the host cells. Here, we summarize the major evasion mechanisms employed by malaria parasites **(Fig 3**).

### Antigen diversity

Malaria antigens such as MSP-1 and -2, apical membrane antigen 1 (AMA1), and circumsporozoite protein (CSP) are highly diverse in parasite populations [30]. The extensive diversity of malarial surface antigens is one of the mechanisms for immune evasion and the main difficulties in developing an effective vaccine (**Fig 3**). Immunity against the erythrocytic stages of the rodent malaria parasite *P*. *c*. *chabaudi* was shown to be strain specific, further supporting an immune escaping mechanism by changing the immune targets [31].

### The variant var, rif, and stevor genes and antigenic variation

The protein encoded by the *var*, *rif*, and *stevor* genes, their binding receptors, and potential functions in parasite biology have been well summarized [32]. In addition to having many gene copies and switching gene expression leads to antigenic variation, the *var* genes encoded protein PfEMP-1 mediates cytoadherence of iRBCs to endothelium and formation of rosettes to avoid clearance by the spleen [32]. They can also suppress cytokine and chemokine production by inhibiting NF-κB signaling in monocytes and macrophages, reduce dendritic cell (DC) antigen-presenting ability by binding to CD36 and CD51 [33], and inhibit cytokine release by NK cells and γδ T cells [34]. Therefore, the *var* genes play diverse roles in immune evasion and suppression.

### RIFIN-mediated inhibition

The *P*. *falciparum* genome also has approximately 150 copies of genes encoding RIFIN (repetitive interspersed families of proteins) expressed on the infected red cell surface [35]. RIFIN can suppress host immune cell activation through direct interaction with host receptors, including Type A erythrocyte antigen, sialic acid on glycophorin A, leukocyte-associated immunoglobulin-like receptor 1 (LAIR1), leukocyte immunoglobulin-like receptor subfamily B 1 (LILRB1), and LILRB2 [36]. LAIR1, LILRB1, and LILRB2 are inhibitory immune receptors, and the binding of RIFIN to LILRB1 on immune cells can suppress IgM production by B cells and reduce cytotoxicity NK cells [36]. Interruption of the interaction between RIFIN and the inhibitory immune receptors may partially release immune suppression and improve host-protective immune responses.

### STEVOR and antigenic variation

STEVORs (subtelomeric variant open reading frame, approximately 30 copies) are variant proteins that are expressed in Maurer’s clefts, the cytosol and membrane of schizont-infected RBCs, and at the apical end of merozoites [37,38]. The expression of different STEVORs on the surface of the iRBC plays a role in the antigenic diversity and agglutination of iRBCs [39]. Malaria parasites infecting rodents and nonhuman primates also have the *Plasmodium* interspersed repeat (*pir*) multigene gene family. The proteins encoded by these genes are expressed on or near the surface of iRBCs and are involved in antigenic variation and immune evasion [40].

### Evasion of complement lysis

Human antibodies were found to activate complement against *P*. *falciparum* sporozoites and were associated with protection against malaria in children [41]. *P*. *falciparum* gamete surface protein PfGAP50 could bind to host complement regulator factor H (FH) and inactivate protein C3b to evade complement-mediated lysis within the mosquito midgut [42]. Similarly, *P*. *falciparum* merozoites recruited FH and C1 esterase inhibitor (C1-INH) to escape complement-mediated lysis [43,44]. More recently, *P*. *falciparum* gametes and sporozoites were shown to hijack plasmin to evade complement attack by degrading C3b [45].

### Hemozoin-mediated immune inhibition

Hemozoin (HZ) is a key product produced by malaria parasites. HZ has been associated with severe malaria anemia (SMA), immunosuppression, and cytokine dysfunction [46]. Macrophages had impaired functions with a long-lasting oxidative burst after ingestion of *P*. *falciparum*-infected erythrocytes or isolated malarial HZ [47]. Differentiation and maturation to DC from HZ-fed monocytes were impaired with a blunted expression of MHC II and co-stimulatory molecules CD1a, CD40, CD54, CD80, and CD83 [48]. HZ-induced DC dysfunction compromised CD4^+^ Tfh function and reduced B cell responses via NLRP3 inflammasome activation [49]. In contrast, malaria HZ could act as a proinflammatory danger signal that activates the NALP3 inflammasome and releases IL-1β [50]. In another study, pure synthetic HZ stimulation of naive murine macrophages activated NF-κB and ERK signaling in naive murine macrophages independent of MyD88 and the NALP3 inflammasome pathway via release of uric acid in vivo [51]. Purified HZ was also found to up-regulate DC maturation with marked increases in cell-surface molecules and IL-12 production [52], which can be explored as an adjuvant to improve vaccine efficacy [53].

### Systematic immune suppression

Malaria-induced immune suppression was reported in a human vaccination study decades ago [54]. In an analysis of gene expression of samples from experimental infection of malaria-naïve volunteers and Cameroonian adults infected with *P*. *falciparum*, 2 to 3 times more genes were repressed than induced compared to uninfected controls [55]. Additionally, up-regulated genes enriched in GO terms of the immune response, inflammatory response, antigen presentation, and other immune pathways were observed in the malaria-naïve volunteers but not in the Cameroonian adults. In a more recent study of controlled malaria infection of Europeans and Africans, preexposed African individuals showed an increase in regulatory T (Treg) cells, ILC2s, and cells expressing PD-1, whereas Europeans had increased classical monocytes and highly up-regulated interferons [56]. Asymptomatic malaria infections also showed immunosuppressive blood transcriptional signature with up-regulation of pathways involved in controlling T-cell function, including the gene encoding cytotoxic T-lymphocyte-associated protein 4 (CTLA-4) [57]. Infections of different *P*. *yoelii* strains or subspecies down-regulated many genes in host immune response pathways such as antigen processing and presentation, DC and monocyte chemotaxis, positive regulation of αβT-cell activation, Th1 activation, DC maturation, and NFAT immune regulation [58].

### Dysfunction and exhaustion of immune cells

The observations of systemic immune suppression indicate dysfunction of immune cells. Adherence of intact *P*. *falciparum*-infected erythrocytes to DCs derived from human peripheral blood cells can inhibit DC maturation and reduce their capacity to stimulate T cells [59]. Blood-stage parasites could inhibit mouse DC maturation and the capacity to initiate CD8^+^ T cell immune responses against the initial liver stage of *P*. *yoelii* parasites [60]. Repeated malaria parasite infection impaired DC function that could affect the generation of helper T cells and B cell responses in animal models and humans [61].

Malaria parasite infection of mice and humans also causes T-cell exhaustion characterized by poor effector functions and the loss of the cells by apoptosis [62]. Increased expression of T cell exhaustion and senescence markers (PD-1, CTLA-4, and CD57) on CD8^+^ and CD4^+^ T cells were observed in the symptomatic children compared to the asymptomatic and healthy controls [63]. Higher frequencies of PD-1-expressing CD4^+^ T cells were found in children in Mali after infection with *P*. *falciparum*, and blockade of PD-L1 and the inhibitory receptor LAG-3 restored CD4^+^ T cell function in mice infected with *P*. *yoelii* but not in those infected with *P*. *chabaudi* [64]. Similarly, PD-1 was expressed in human CD8^+^ T cells from individuals infected with malaria parasites [65] and mediated the loss of parasite-specific CD8^+^ T cells during the acute phase of *P*. *chabaudi* malaria in mice [66]. Therefore, a blockade of PD-1 will improve CD8 T cell function and protect against chronic malaria [62]. Atypical memory B cells (atMBCs) characterized by the expression of exhaustion markers with reduced B cell receptor signaling and effector function were also observed in malaria patients [67], but the roles of atMBCs in immune inhibition require additional investigation.

*Plasmodium* DNA, HZ, or extracellular vesicles can impair the function of monocytes and macrophages [68]. Malaria parasite infection induced macrophage dysfunction in *P*. *yoelii* 17XNL-infected C57BL/6j mice, leading to SMA [25]. Monocytes loaded with HZ could suppress erythropoiesis in the bone marrow through IFN-γ-mediated apoptosis of the erythroid progenitors and antagonized GATA1 transcriptional activity in *P*. *cynomolgi*-infected rhesus macaques [69]. *P*. *falciparum* infection polarized and accumulated M1 macrophages in the lungs of patients [70]. SMA patients had a greater proportion of monocytes loaded with HZ than controls, and children with severe malaria showed a lowered expression of TLR2 and TLR4, which correlates with monocyte inactivation [71].

RIFIN on the surface of human iRBCs could bind to the inhibitory receptors LILRB1 or LAIR1 on NK cells and reduce NK cell cytotoxicity [72]. Malaria parasite infection also induced neutrophils with reduced oxidative burst activity in Gambian children, which correlated significantly with markers of hemolysis and HO-1 induction [73].

### Modulation of immune signaling pathways

CTLA-4 and PD-1 are negative checkpoint regulators of T cell immune function, and inhibition of these targets is being used as immunotherapies for various cancers. The activation of T cells requires the interaction of the T cell receptor (TCR) and CD28 with MHC II and B7 co-stimulatory molecules (CD80 and CD86) located on the antigen-presenting cells, respectively [74]. The interaction of CTLA-4 with the B7 molecules initiates an inhibitory signal, which can be blocked (checked) by CTLA-4 inhibitors. Similarly, the interaction of PD-1 and PD-L1 leads to the negative regulation of T cells, which can be blocked by inhibitors blocking the interaction [74]. CD80 and CD86 do not bind to T cell receptors with the same affinity or effect [75]. CD86 is important for initiating immune responses, whereas CD80 might dampen immune responses after binding to CTLA-4 or PD-L1 [76]. DCs expressing CD80, but not CD86, could significantly suppress lymphocyte proliferation and increase production of IL-10, TGF-β, and IDO [77].

The PD-1- and CTLA-4-mediated immune inhibition pathways also play an important role in the host response to malaria parasite infections [78]. Higher T cell activation and IFN-γ production levels were observed after antibody-mediated blockade of the CTLA-4 or PD-1 pathways during PbA-infection in the BALB/c mice. PD-1-deficient mice could rapidly and completely clear *P*. *chabaudi* infections by increasing parasite-specific CD8^+^ T cells [66]. Persistent *P*. *falciparum* exposure was associated with an increased frequency of CD4^+^ T cells expressing PD-1 alone and PD-1 in combination with lymphocyte-activation gene-3 (LAG-3) [65]. Similarly, increased CD4^+^ T cells expressing CTLA-4, OX40, GITR, TNFRII, and CD69 were found in patients acutely infected with *P*. *vivax*, *P*. *falciparum*, or both [79].

MARCH1 (or MARCHF1) is an E3 ubiquitin ligase and immune regulator that regulates protein levels of CD86, MHC II (HLA-DR alpha and beta), TFRC, FAS, and key molecules (MAVS and STING) in IFN-I pathways [80,81]. MARCH1 deficiency increased CD86^+^ DC populations and IFN-γ and IL-10 levels in C57BL/6j mice at day 4 after infection with *P*. *yoelii* YM, leading to improved host survival [81]. The functions of mDCs were compromised by *P*. *falciparum* exposure, with impaired HLA-DR and CD86 expression and effector T cell cytokine responses [82]. An early IFN-I response helps parasitemia control, but chronically elevated levels of IFN-I in later infection may impair adaptive immune response and increase disease severity [83]. CD83 could block IL-10-driven, MARCH1-dependent ubiquitination and degradation of MHC II and CD86 in DCs [84], which may be explored for immunotherapy. There are more immune regulatory molecules, such as SOCS1 and RTP4, that can affect malaria parasite infections [29,85,86]; due to space constraints, we cannot discuss them individually here.

### Epitope masking

Antibody avidity and titer after vaccination were associated with increased levels of vaccine efficacy. However, induction and maintenance of high antibody titers above a protective threshold have proven challenging [87]. In one study, CSP-specific antibodies and B cells expanded in humans and Ig-knockin mice following the first and second immunization with PfSPZ, but not after the third dose, which might be mediated through epitope masking via the feedback of circulating anti-CSP antibodies [88]. Indeed, invasion-blocking antibodies were induced by a CSP N-terminal polypeptide of 99 amino acids only when it was presented in the absence of the rest of the protein [89]. Structural-based design of the mature and infection-relevant forms of antigens may improve vaccine efficacy [90].

## 5. Modulation of immune response to increase vaccine efficacy

To deal with antigen diversity and antigenic variation, much effort has been made to find conserved and protective epitopes for vaccine development. RTS,S/AS01 and R21 are the 2 vaccines approved by WHO. The vaccines are designed based on the “conserved” repeat region of the CSP protein to avoid epitope allelic variation and can provide partial protection [91]. Another approach is to use whole parasites, either sporozoites or asexual stages, as antigens, which has achieved promising results [92,93]. Whole parasite vaccines consist of multiple unknown antigen targets that may generate protective antibodies against more than 1 parasite strain infection. Various efforts have been employed to enhance molecular vaccine efficacy or increase antibody titer. The R21 vaccine used a single virus-like CSP-hepatitis B surface antigen (HBsAg) fusion protein with a much higher proportion of CSP than in the RTS,S, leading to a minimal antibody response to the HBsAg carrier and improved vaccine efficacy [94]. Engineered chimeric antigens of the AMA1 DII loop with RON2L enhanced antibodies targeting conserved epitopes on AMA1 and increased neutralization of nonvaccine type parasites [95]. However, these and other efforts to increase vaccination efficacy or antibody titers by refining epitopes and/or antigen presentation with known adjuvants may not be sufficient to overcome the immune inhibition induced by prior parasite exposure or the presence of parasites in the patient’s blood. Modulation of host immune responses may be required to overcome malaria-induced inhibition. Modulation of host metabolism or immune response to treat malaria has been reported previously. Administration of the glutamine analog 6-diazo-5-oxo-L-norleucine (DON), CD47, or interleukin-15 complex rescued mice from PbA ECM [96–98].

**Fig 4:**
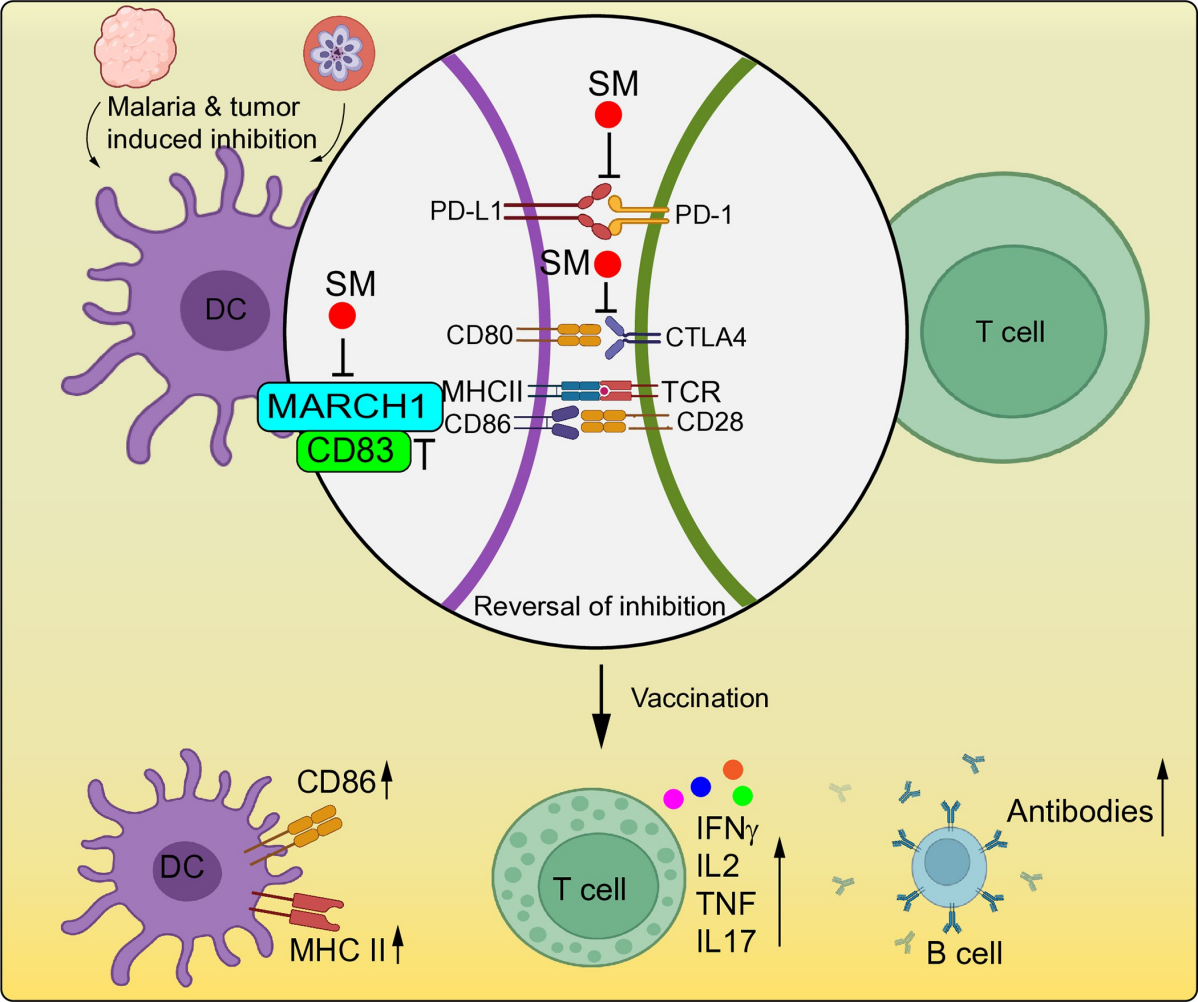
Potential strategies to reverse immune inhibition and enhance vaccination efficacy. Inhibition of interactions of PD-1/PD-L1 and CTLA-4/CD80 with SMs can block the immune checkpoint inhibition pathways to activate T cells. Additionally, inhibition of MARCH1 using SMs or specific peptides from CD83 may increase levels of CD86 and MHC II expression on DCs, promoting T cell activation. Combining monoclonal antibodies and SMs blocking PD-1/PD-L1 interaction or inhibiting MARCH1 may achieve better results than individually blocking PD-1/PD-L1 or inhibiting MARCH1. Enhancing T cell activities will promote antibody production by B cells, leading to better vaccination efficacy. Some images of cells and receptors were adopted from BioRender.

The expression of MHC II and the co-stimulatory molecule CD86 on DCs is required to activate T and B cells to produce antibodies. The key issue in increasing vaccine efficacy may be enhancing the expression of CD86/MHC II on antigen-presenting cells before vaccination, although whether enhancing CD86/MHC II expression can totally reverse malaria-induced immune inhibition remains to be tested. Postponing vaccination until a malaria infection has been cleared to “release” immune inhibition may be a good strategy for vaccinating populations in endemic areas where most individuals have been exposed to malaria multiple times [99]. Screening and testing small molecules (SMs) and peptides to block immune inhibition is becoming an emerging field in treating cancers and other diseases. SMs can also be explored to improve malaria vaccine efficacy by blocking various immune inhibition pathways and/or host–parasite molecular interactions (**Fig 4**). Vaccinating individuals with multiple prior exposures or active parasite infection could be like driving a car with the handbrake on.

### SM immune checkpoint modulators

Malaria is a curable disease, and artemisinin combination therapies are still effective [100]. Immunotherapies such as PD-1/PD-L1 blockade using monoclonal antibodies, developed for treating cancers, will not be practical for malaria treatment or activation of host immune response before vaccination, mainly due to high costs and potential immune-related adverse events (irAEs). SMs are being developed as checkpoint blockers with advantages over antibody treatment, including lower cost, easier dosing (oral), and better management of irEAs [101]. Antibodies typically have a relatively long half-life in vivo, and in the event of irAEs such as cytokine storm, corticosteroids are used to suppress inflammation, which may, in turn, put the patients at a higher risk of developing infections. SMs generally have a shorter half-life than antibodies and may be synthesized at lower costs. A novel series of [1,2,4]triazolo[4,3- a]pyridines were found to be potent inhibitors of the PD-1/PD-L1 interaction, particularly compound A22 having an IC_50_ of 92.3 nM in blocking PD-1/PD-L1 interaction [102]. Further structural refinement improved IC_50_ values to 3.0 to 25 nM [103]. Interestingly, screening random phage libraries for peptides bound to the recombinant human PD-1 identified potential PD-1 checkpoint inhibitors that could be used as adjuvants for vaccines against infectious diseases [104]. In a follow-up study, a 22-amino acid immunomodulatory peptide derived from a *Bacillus* bacterium (LD01) with similarity to a PD-1 peptide antagonist was a potent immunomodulator that could stimulate T cell responses [105]. When combined with an adenovirus-based or irradiated sporozoite-based malaria vaccine, LD01 significantly enhanced antigen-specific CD8^+^ T cell expansion. In a study using an AI (artificial intelligence) algorithm to screen a library of approximately 10 million compounds for SMs recognizing a putative binding pocket on CTLA-4, several lead SMs were found to bind to CTLA-4 and inhibit its interaction with CD80 [106]. Compounds bound to CTLA-4 at low micromolar concentrations could inhibit tumor development in hCTLA-4 mice at 5 to 25 mg/kg dosages.

### Blockade of MARCH1 degradation of CD86 and MHC II

MARCH1 is a potent immune regulator that can be explored for immune therapies. MARCHI transduced DCs secreted high levels of IL-10 after LPS stimulation and stimulated T cells toward the Treg subset [107]. The TM domain of CD83 could enhance MHC II and CD86 expression by blocking MHC II association with MARCH1 [84]. CD83 may be a promising immune modulator with therapeutic potential, and soluble CD83 can be explored to treat various mouse models of autoimmune and inflammatory diseases [108]. Alternatively, SMs can be identified as inhibitors of MARCH1 activity, although no SM MARCH1 inhibitors have been reported.

### Safety concerns on immune therapies

Immune checkpoint blockade therapies are often associated with a spectrum of side effects, with various onsets described for the different toxicities such as cytokine storm [109]. To reduce the chance of inducing irEAs, immune modulators and vaccination can be administered during the dry season when an individual has no active infection and strong inflammation. The SM dosage, half-life in vivo, and the timing of administration will have to be tested in clinical trials before field application.

## 6. Conclusions

Malaria is a complex disease with a spectrum of symptoms determined by various factors derived from host–parasite genetic interactions. Rodent and nonhuman primate malaria parasites are good models to dissect disease and immune mechanisms. Results from animal models can then be tested in human malaria patients. Clinical association studies are confounded by many unknown or uncontrollable conditions such as genetic variations in the host and parasite, mix-infection, unknown timing of infection, prior exposure, etc. Historically, most studies on host immunity focused on up-regulated genes in response to malaria parasite infections. It is time to pay more attention to the immune pathways inhibited in malaria. Measures to release immune inhibition will be critical to improve vaccine efficacy. Searching and testing SMs to block immune checkpoints (PD-1/PD-L1, for example), as is being done in anti-cancer therapies, may provide a fruitful approach to enhance vaccine efficacy. MARCH1 and CD83, in addition to PD-1 and CTLA-4, are promising immune regulators that can be explored to activate host immune responses, including activation of T-helper cells and production of IFN-I that appear to be associated with successful immunotherapy for cancers [110]. Finally, it will be critical and challenging to find SMs and dosages that can activate the host immune response to enhance vaccine efficacy without triggering a cytokine storm or irAEs.
